# Performance of large language models in mitral valve surgery patient education: a comparative analysis

**DOI:** 10.1186/s12911-026-03662-3

**Published:** 2026-06-29

**Authors:** Banu Bahriye Akdag, Mehmet Senel Bademci, Ihsan Peker, Guven Okay Karaca, Cagrı Kandemir, Barcin Özcem, Huseyin Durmaz, Meryem Cakır, Irem Ozcetin, Hidayet Onur Selcuk

**Affiliations:** 1 Department of Cardiovascular Surgery, University of Health Sciences, Izmir City Hospital, Izmir, 35530 Turkey; 2 Department of Cardiovascular Surgery, Izmir City Hospital, Izmir, 35530 Turkey; 3 Department of Cardiovascular Surgery, Faculty of Medicine, Near East University, Nicosia, 99138 Cyprus; 4 Department of Cardiovascular Surgery, Konya City Hospital, Konya, 42020 Turkey; 5 Department of Family Medicine, Izmir City Hospital, Izmir, 35530 Turkey; 6 Department of Cardiovascular Surgery, Atatürk Training and Research Hospital, Izmir Katip Çelebi University, Izmir, 35360 Turkey

**Keywords:** Large language models, Artificial intelligence, Mitral valve surgery, Patient education, Accuracy, Readability

## Abstract

**Background:**

Large language models (LLMs), a form of artificial intelligence, are increasingly being utilized in healthcare to support patient education and information delivery. The aim of this study was to perform a comparative analysis of five different LLMs (i.e., ChatGPT-4o, Claude 3.7 Sonnet, Gemini 2.5 Pro Preview, DeepSeek-V3, and Microsoft Copilot) in terms of accuracy, completeness, and readability, based on their responses to frequently asked questions in preoperative patient education for mitral valve surgery (MVS).

**Methods:**

A standardized questionnaire comprising seven frequently asked questions by patients prior to MVS was developed. Prompting procedures and model parameters were fully reported to support reproducibility. These questions were presented to each LLM in an identical manner. The responses were evaluated by two academic experts in cardiac surgery using structured assessment criteria across three main dimensions: accuracy, completeness, and readability. For the readability analysis, the Simplified Measure of Gobbledygook (SMOG) Index and the Flesch-Kincaid Grade Level (FKGL) scale were utilized.

**Results:**

The ChatGPT-4o and Gemini 2.5 Pro Preview models received statistically significantly higher scores than Claude 3.7 Sonnet and Microsoft Copilot for both accuracy (median 5 for ChatGPT-4o and Gemini 2.5 Pro Preview vs. 4 for Claude 3.7 Sonnet and Microsoft Copilot, p < 0.001) and completeness (median 5 for Gemini 2.5 Pro Preview vs. 3 for Claude 3.7 Sonnet, p < 0.001). Claude 3.7 Sonnet achieved the highest readability scores, with significantly lower SMOG (10.90 for Claude 3.7 Sonnet vs. 12.24 for ChatGPT-4o, *p* = 0.006) and FKGL (8.0 for Claude 3.7 Sonnet vs. 9.04 for ChatGPT-4o, *p* = 0.004) scores, indicating simpler and more comprehensible sentence structures. Significant differences were observed among the evaluated models across all three assessment dimensions (p < 0.001 for all comparisons).

**Conclusions:**

The LLMs represent valuable supplementary tools in patient education processes. However, their implementation in clinical practice must be carefully evaluated, particularly with regard to accuracy and completeness. This study highlights the potential applicability of ChatGPT-4o and Claude 3.7 Sonnet models for preoperative patient education in MVS, while emphasizing that all LLMs should be used under the supervision and guidance of healthcare professionals. For LLMs to be reliably utilized in the medical field, improvement in medical accuracy and standardization are essential.

**Supplementary Information:**

The online version contains supplementary material available at https://doi.org/10.1186/s12911-026-03662-3.

## Introduction

Mitral valve diseases are among the most common valvular heart pathologies, leading to significant clinical outcomes. These conditions are classified into two main clinical forms: mitral stenosis and mitral regurgitation. Over time, they may impair left ventricular function and predispose patients to heart failure, atrial fibrillation, and thromboembolic events [1]. In symptomatic patients, medical therapy provides limited benefits, and surgical intervention often offers a more definitive solution. Currently, mitral valve repair is usually preferred over replacement, particularly in degenerative mitral regurgitation, owing to its certain advantages in preserving valve function, maintaining left ventricular geometry, and improving survival. Furthermore, advancements in surgical techniques and the widespread adoption of minimally invasive approaches have shortened postoperative recovery times and reduced complication rates [2].

The decision to undergo MVS involves not only anatomical and clinical factors, but also the patient’s preferences, lifestyle, expectations, and understanding of potential outcomes. Comprehensive patient education and information-sharing processes led by heart teams have been shown to improve patient engagement and positively influence satisfaction with care [2].

The success of MVS heavily relies on effective patient education, which is crucial for treatment adherence and complication reduction. Clearly communicating the differences between valve repair and replacement and, if replacement is necessary, between bioprosthetic and mechanical valves, as well as the potential risks and benefits, supports patients in actively participating in informed decision-making. Current guidelines recommend a patient-centered approach as a cornerstone of surgical success and emphasize the ethical and clinical importance of conducting the informed consent process with great care [1, 2].

Large language models (LLMs), a subset of artificial intelligence and machine learning, have emerged as powerful tools in surgical practice, providing human-like textual responses to clinical queries and patient education needs. In subsequent years, numerous LLMs have achieved widespread use, including ChatGPT-4o by OpenAI, Claude 3.7 Sonnet by Anthropic, Gemini 2.5 Pro Preview by Google, Microsoft Copilot, and DeepSeek-V3. These models can provide rapid, accessible, and readable basic-level information on topics such as preoperative procedures, surgical risks, alternative treatments, and the recovery process.

Recent studies have shown that certain LLMs may deliver incomplete or misleading information regarding surgical risks and postoperative recovery [3]. Nonetheless, their ability to communicate information with high readability and in non-technical language represents a significant advantage, particularly for individuals with low health literacy.

Currently, healthcare professionals view these technologies as supportive tools in patient education; however, it is strongly emphasized that final medical decisions must always be made by clinical experts. In recent years, AI-based health communication has increasingly become a complementary component of patient-centered care in modern medicine.

Large language models (LLMs) are also being utilized for candidates undergoing cardiac surgery [4]. In particularly complex procedures such as MVS, these models can facilitate rapid access to information, enhance physician–patient communication, and support patients’ psychological preparedness for the surgical process [5]. However, despite their growing use, there is limited research comparing the accuracy, completeness, and readability of the information these models provide specifically for MVS patient education. This gap makes it challenging to determine which models offer the most reliable and accessible information to support patient understanding and decision-making.

## Materials and methods

### Study design

This study was designed as a comparative evaluation of multiple large language models (LLMs) in the context of preoperative patient education for mitral valve surgery (MVS).

#### Question source, prompting procedure and model access

A topic-primed, zero-shot prompting approach was used. At the beginning of each session, a brief topic-priming statement was provided to the model in the form: “The following are patient-oriented questions about mitral valve surgery; please answer each question accordingly.” This single contextual cue was used to establish the clinical topic of the questions, which—being derived verbatim from a patient information page—did not themselves contain explicit references to mitral valve surgery. Apart from this topic-priming statement, no additional descriptive text, role-based prompt, prompt-engineering technique, or contextual information from the source website was provided. Each of the seven questions was then submitted independently as a standalone query within the same session, with no follow-up prompts, clarifications, or interactive exchanges. This protocol was intentionally chosen to reflect a realistic patient-information scenario in which a user opens a chat with a brief framing of their clinical situation and then asks short, generic questions. The evaluation was based on a standardized set of seven open-ended questions commonly asked by patients prior to mitral valve surgery. The questions were derived verbatim from the patient information section of the Society for Cardiothoracic Surgery in Great Britain & Ireland, available at: [https://scts.org/patients/heart/procedures/20/mitral_valve_surgery]. The website link is provided solely to document the source of the question wording; no accompanying explanatory text from the website was included in the prompts. This approach was intentionally chosen to evaluate each model’s ability to generate responses based solely on its pre-trained knowledge, beyond the brief topic-priming statement described above, reflecting real-world scenarios in which patients may open a chat with a short framing of their clinical situation and then pose questions directly without providing any further clinical context. No modifications were made to the wording of the questions.

The exact prompts used were:

1. Why do I need surgery?
2. What does the anaesthetic involve?
3. What does the surgery involve?
4. What happens after surgery?
5. What are the benefits and risks?
6. What should I do when I go home?
7. What are the alternatives to surgery?

All models were accessed through their publicly available, web-based user interfaces. The evaluated models included ChatGPT-4o (OpenAI), Claude 3.7 Sonnet (Anthropic), Gemini 2.5 Pro Preview (Google), DeepSeek-V3, and Microsoft Copilot, as available in April 2025. No application programming interfaces (APIs), custom integrations, or third-party tools were used. No system-level instructions, role-based prompts, or prompt engineering techniques were used. All models were queried using the default generation parameters provided by their respective platforms; no manual adjustments were made to temperature, top_p, or maximum token limits. No system-level modifications were applied. Each question was submitted in a new chat session to prevent contextual carryover between prompts, and a single response per question was collected for analysis. The queries were performed using the web-based interfaces of the respective platforms in April 2025, with web browsing or external search features disabled during response generation. Microsoft Copilot was accessed via its publicly available web interface. As Copilot functions as an interface that may be powered by different underlying large language models depending on system configuration and updates, the exact model version cannot be definitively specified.

#### Output capture and preparation

No edits or content changes were made on model responses before evaluation. Only minor formatting adjustments were applied to standardize the appearance of the responses during scoring.

### Selection of LLM

Language models which were freely or partially freely accessible to any user were selected for the study. Additionally, the selected models were chosen based on their popularity and accessibility within the healthcare sector. This approach was intended to reflect real-world patient access to AI-generated health information.

#### Evaluation process

Two experts with at least 10 years of experience in MVS independently evaluated the data in terms of accuracy and completeness using a five-point Likert scale to maintain objectivity. For the accuracy evaluation, current guidelines on MVS and the clinical expertise of specialists in the field were used as primary reference sources [1, 2]. After the initial independent evaluations, the evaluators met to reach a consensus and determine a final, unified score. To assess inter-rater reliability, the intraclass correlation coefficient (ICC) was calculated [6], and the result was found to be 0.85, indicating high reliability.

To standardize the scoring process, predefined operational criteria were used for both accuracy and completeness assessments. Accuracy scores were assigned by comparing the content of each LLM response with current international mitral valve surgery guidelines and established clinical references. Responses were evaluated based on factual correctness, consistency with guideline-based recommendations, and absence of misleading or incorrect statements.

Completeness scores were assigned based on whether the response adequately covered key domains of patient education, including surgical indications, procedural description, risks and complications, alternative treatment options, and postoperative expectations.

To minimize potential evaluation bias, all responses were anonymized prior to assessment. The evaluators were blinded to the identity of the language model that generated each response. Responses were presented in randomized order and independently scored by both reviewers before consensus scoring was performed.

#### Evaluation criteria

##### Accuracy evaluation

A five-point Likert scale was used to evaluate the accuracy of the information [7]: 5 points=Completely accurate, 4 points=More accurate than inaccurate, 3 points=Accuracy and inaccuracy are roughly equal, 2 points=More inaccurate than accurate, 1 point=Completely inaccurate.

##### Completeness evaluation

The completeness of the information was also evaluated using a five-point Likert scale [7]: 5 = Completely sufficient (Fully covers the topic/No missing information), 4 = Mostly sufficient (Covers the topic/Minor omissions), 3 = Moderately sufficient (Relevant to the topic/Some ambiguities), 2 = Mostly insufficient (Partially related to the topic/Significant omissions), 1 = Very insufficient (Unrelated to the topic/Overly basic or nonsensical). Accuracy and completeness assessments were based on concordance with current international guidelines for mitral valve surgery and the clinical judgment of the evaluating surgeons. No predefined or model-generated “gold standard” reference text was used; instead, evaluations relied on guideline consistency and expert consensus.

##### Readability evaluation

To evaluate the readability of the responses, the Simplified Measure of Gobbledygook (SMOG) Index [7] and the Flesch-Kincaid Grade Level (FKGL) scale were used [8]. The website https://readabilityformulas.com/readability-scoring-system.php was used for readability calculations.

### Statistical analysis

Statistical analysis was performed using the SPSS for Windows version 23.0 software (IBM Corp., Armonk, NY, USA). Comparisons of the LLMs in terms of accuracy and completeness were conducted using the Kruskal-Wallis test, followed by *post-hoc* Dunn-Bonferroni tests after detecting overall significance. The normality of the readability scores was checked using the Shapiro-Wilk test. Continuous variables were presented in mean ± standard deviation (SD) or median (min-max), while categorical variables were presented in number and frequency. The readability scores which met the normality assumption were compared using one-way analysis of variance (ANOVA). Subsequent subgroup analyses following ANOVA were performed using the Bonferroni corrections. A *p* value of < 0.05 was considered statistically significant.

## Results

**Fig. 1:**
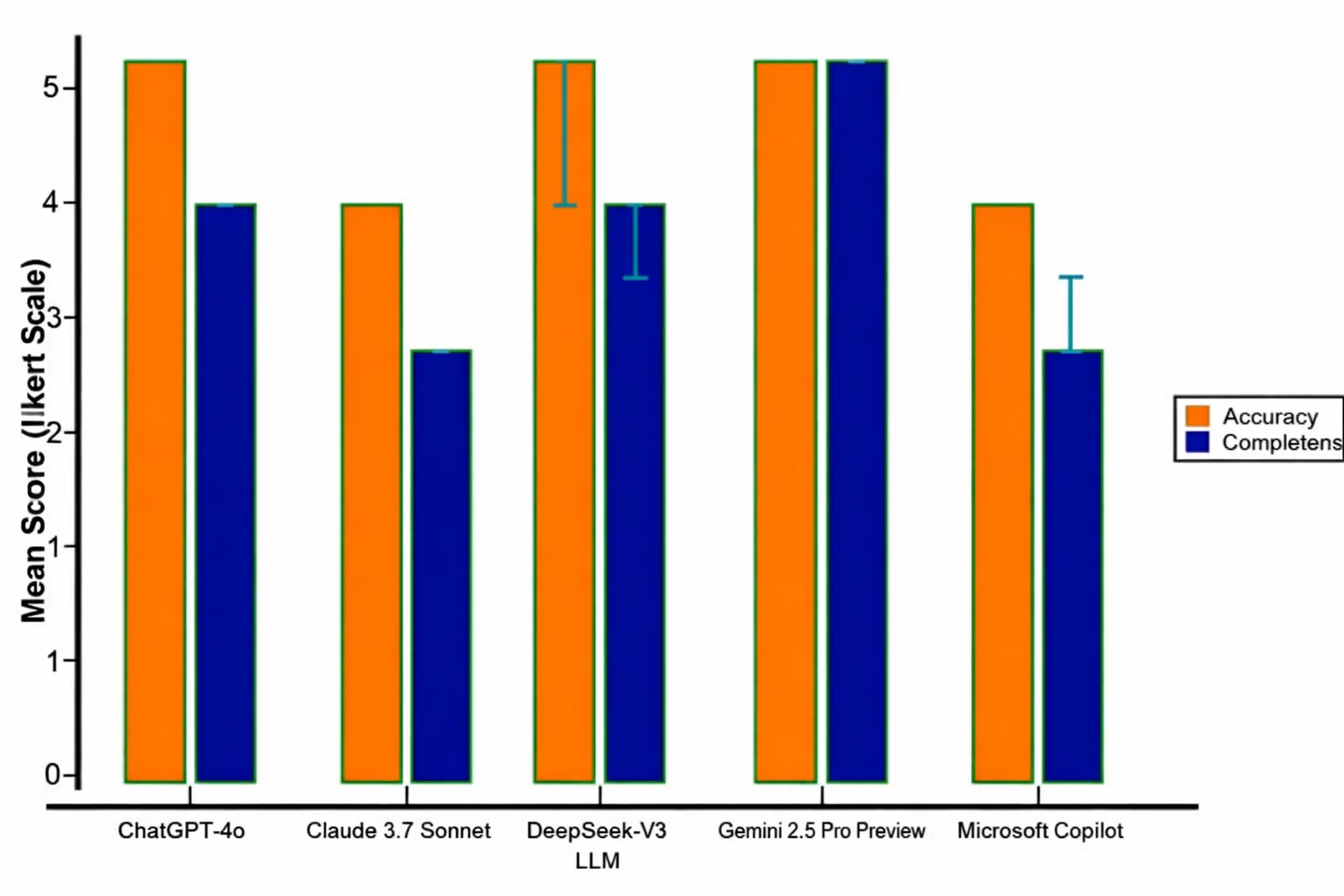
Comparison of accuracy and completeness scores across large language models (LLMs) in patient education on mitral valve surgery. (Likert scale)

A statistically significant difference was found among the LLMs in terms of accuracy scores (p < 0.001) (Table 1). In the *post-hoc* analyses, the accuracy level of ChatGPT 4o was found to be significantly higher than that of Claude 3.7 Sonnet (p = 0.002) and Microsoft Copilot (p = 0.002). Similarly, Gemini 2.5 Pro Preview had a higher accuracy level compared to Claude 3.7 Sonnet (p = 0.002) and Microsoft Copilot (p = 0.002). While the pairwise comparisons reported above reached statistical significance, other pairwise comparisons not specifically mentioned did not demonstrate significant differences (p > 0.05) (Fig. 1).

**Table 1:** Comparative performance of large language models in accuracy, completeness, and readability metrics

< LLM Model	Accuracy	Completeness	SMOG	FKGL
ChatGPT 4o	5 (5–5)	4 (4–4)	12.24 ± 0.69	9.04 9.04 ± 0.55
Claude 3.7 Sonnet	4 (4–4)	3 (3–3)	10.90 ± 0.54	8.0 ± 0.41
DeepSeek-V3	5 (4–5)	4 (3–4)	12.61 ± 0.72	9.37 ± 0.53
Gemini 2.5 Pro Preview	5 (5–5)	5 (5–5)	12.41 ± 0.64	9.17 ± 0.48
Microsoft Copilot	4 (4–4)	3 (3–4)	11.84 ± 0.66	8.77 ± 0.49
p-value	**< 0.001**	**< 0.001**	**< 0.001**	**< 0.001**

Data are presented as median (minimum–maximum) for Accuracy and Completeness scores, and as mean ± standard deviation for Flesch-Kincaid Grade Level (FKGL) and Simplified Measure of Gobbledygook (SMOG) scores

There was a statistically significant difference among the LLMs regarding completeness scores (p < 0.001). In the *post-hoc* analyses, the completeness score of Gemini 2.5 Pro Preview was found to be significantly higher than that of Claude 3.7 Sonnet (p < 0.001) and Microsoft Copilot (p < 0.001). In addition to the significant comparisons reported above, other pairwise comparisons did not reach statistical significance (p > 0.05). (Fig. 1)

**Fig. 2:**
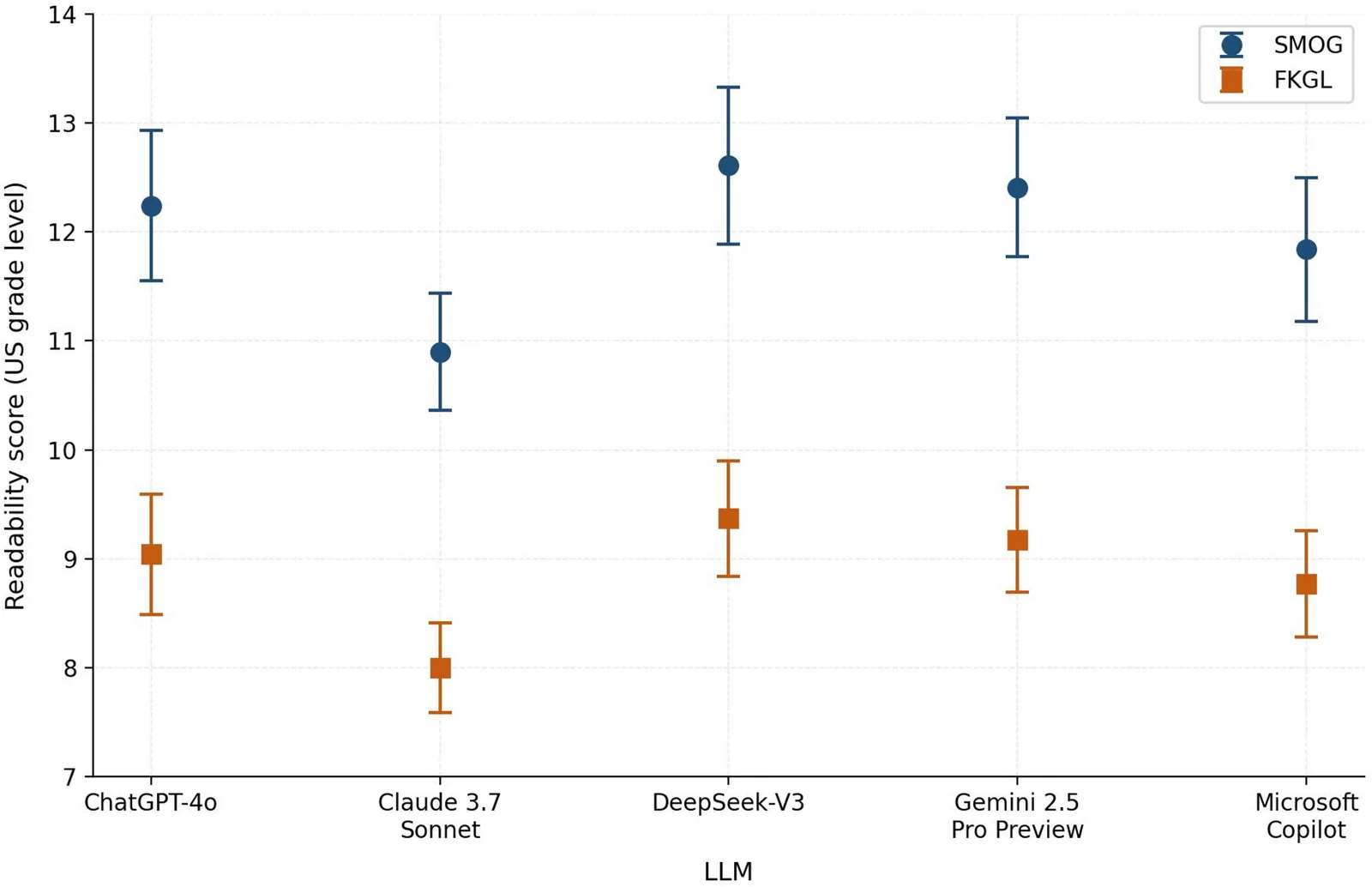
Comparison of readability scores (SMOG and FKGL) across LLMs in patient education on mitral valve surgery. The y-axis represents the readability score, expressed as US grade level (higher values indicate text requiring a higher level of education to comprehend). Data are shown as mean ± standard deviation

Analysis of the SMOG scores revealed a statistically significant difference among the LLMs, consistent with the values presented in Table 1. (p < 0.001). In the *post-hoc* analyses, the mean SMOG score of Claude 3.7 Sonnet was found to be significantly lower than that of ChatGPT 4o (10.90 & 12.24; *p* = 0.006), DeepSeek-V3 (10.90 & 12.61; p < 0.001), and Gemini 2.5 Pro Preview (10.90 & 12.41; *p* = 0.001). While the comparisons between Claude 3.7 Sonnet and the other three models were statistically significant as reported, comparisons among ChatGPT-4o, DeepSeek-V3, Gemini 2.5 Pro Preview, and Microsoft Copilot did not demonstrate significant differences (p > 0.05) (Fig. 2).

There was a statistically significant difference in the FKGL scores among the LLMs (p < 0.001). In the *post-hoc* analyses, the mean FKGL score of Claude 3.7 Sonnet was found to be significantly lower than that of ChatGPT 4o (8 & 9.04; *p* = 0.004), DeepSeek-V3 (8 & 9.37; p < 0.001), and Gemini 2.5 Pro Preview (8 & 9.17; *p* = 0.001). Similarly, comparisons among ChatGPT-4o, DeepSeek-V3, Gemini 2.5 Pro Preview, and Microsoft Copilot did not reach statistical significance (p > 0.05). (Fig. 2)

## Discussion

Despite the increasing integration of LLMs into healthcare communication, particularly for patient education, there remains a significant gap in comparative evaluations of these tools in the context of complex surgical procedures. Most existing studies have focused on the performance of individual LLMs or their application in general health information dissemination. However, comprehensive analyses assessing the accuracy, completeness, and readability of multiple, state-of-the-art LLMs in delivering patient-centered information for specific and high-risk interventions, such as MVS, are still limited. In the present study, we evaluated the accuracy, completeness, and readability levels of five LLMs (i.e., ChatGPT 4o, Claude 3.7 Sonnet, DeepSeek-V3, Gemini 2.5 Pro Preview, and Microsoft Copilot), used for patient education and information regarding MVS. Notably, the ChatGPT 4o and Gemini 2.5 Pro Preview models were found to be statistically superior to Claude 3.7 Sonnet and Microsoft Copilot in terms of both accuracy and completeness. Considering the readability of responses generated by the LLMs, the Claude 3.7 Sonnet model yielded statistically more readable responses compared to ChatGPT 4o, DeepSeek-V3, and Gemini 2.5 Pro Preview. Given the limited question set, however, these post-hoc comparisons should be interpreted with caution and viewed as exploratory rather than definitive indicators of model superiority.

In recent years, there has been a growing number of studies examining the accuracy of data generated by LLMs, particularly in the context of patient education. Lee et al. [9] showed that the ChatGPT model yielded higher accuracy in their study on atrial fibrillation patient education. Similarly, other studies have demonstrated that the ChatGPT model provides clinically accurate information at a high level [10]. In a related study by Zhao et al. [11], which focused on developing educational materials for patients with lower back pain, the Gemini 2.5 Pro Preview model achieved similarly high levels of accuracy and completeness as ChatGPT 4o.

Building on these comparative findings, it is important to note that the superior performance of ChatGPT-4o and Gemini 2.5 Pro Preview in terms of accuracy and completeness reflects empirical observations in the present study, but the underlying mechanisms responsible for these differences were not investigated. One potential explanation discussed in the literature involves Retrieval-Augmented Generation (RAG), a framework in which language models access external, domain-specific knowledge sources during response generation [12]. Previous studies have shown that RAG-augmented systems may demonstrate improved factual accuracy and completeness compared to conventional models that rely solely on pre-trained knowledge [13]. However, RAG was not implemented or directly evaluated in the present study. The performance patterns observed here particularly with regard to accuracy and completeness are conceptually consistent with outcomes reported for RAG-augmented deployments in prior research, but this does not imply that RAG was used by the evaluated models, nor does it establish a causal relationship between model architecture and observed performance. These findings should be interpreted as descriptive comparisons rather than mechanistic explanations. Future research employing controlled RAG implementations would be necessary to substantiate any such claims.

Supporting this interpretation, Woo et al. [13] demonstrated that the integration of RAG into large language model workflows significantly improved accuracy in the context of anterior cruciate ligament (ACL) injury information, reporting an average accuracy increase of 39.7%. The RAG framework has therefore been highlighted as a valuable approach for enhancing the reliability of patient education materials compared with traditional LLM-only systems [13]. Given ongoing concerns regarding the potential for LLMs to generate inaccurate or outdated information in medical contexts [14], all outputs in the present study were independently reviewed by two experienced surgeons specializing in the relevant field to mitigate this risk.

Another critical aspect of the data obtained from LLMs is ensuring ease of readability and comprehension. Review of the recent literature reveals numerous studies presenting varying perspectives on this issue [15, 16]. The coexistence of plain language and medical accuracy is of utmost importance for patient safety. In our study, the Claude 3.7 Sonnet model demonstrated the highest performance in terms of readability compared to the other models. In a study conducted by Zhang et al. [17] on inflammatory bowel disease, readability scores were found to be higher for the Claude 3.7 Sonnet model than for ChatGPT-4o and Gemini 2.5 Pro Preview. This finding highlights Claude’s success in generating user-friendly and more comprehensible texts. This advantage stems from the model’s ability to use shorter sentences and less complex vocabulary, which enhances the readability of the generated content, particularly in medical contexts where such language improves overall understanding [16]. In the present study, this advantage was quantified by the significantly lower SMOG and FKGL scores obtained by Claude 3.7 Sonnet. Consistent with this, Jin et al. [18] reported that fast, internally consistent answers make texts clearer and easier to understand.

Furthermore, there are also ethical concerns regarding the use of LLMs in patient education. Patient privacy and data security are of paramount importance in this context. The primary concern relates to the handling of sensitive patient information and avoiding entering it into LLM systems during user interaction [19]. Additionally, there is a risk that patients may accept information generated by the model without questioning it (overtrust), potentially replacing professional medical advice. Therefore, the use of LLMs in patient education should be conducted under strict supervision, transparency, and ethical guidelines. In clinical practice, this implies that LLM-generated content should be used only as an adjunct to clinician-led communication. Patients should be informed when AI-generated information is involved, accompanied by appropriate disclaimers, and such content should be regularly reviewed and updated in line with current clinical guidelines.

### Clinical implications

By improving the clarity and accessibility of medical information, LLMs may contribute to enhanced health literacy and support informed patient understanding. Their potential utility may be greater in busy clinical settings where time constraints limit extensive patient education. However, any integration of LLMs into patient-facing contexts requires appropriate clinical oversight to ensure the accuracy, relevance, and appropriateness of the information provided. Additionally, patient-specific factors such as age, educational background, and digital literacy should be considered when evaluating the feasibility of LLM-assisted educational tools. Within these limitations, LLMs may function as supportive adjuncts, rather than substitutes, in patient communication and shared decision-making processes, and should be used only under direct clinician oversight.

A key methodological contribution of this study is the use of a standardized, zero-shot prompting framework applied uniformly across all evaluated models. By submitting identical patient-oriented questions to each LLM under controlled conditions, we ensured that observed differences in accuracy, completeness, and readability could be attributed to the models themselves, rather than to variations in question phrasing, contextual priming, or interaction style. Detailed reporting of the prompting protocol, model access procedures, and generation parameters was prioritized to support transparency and reproducibility, which are essential for meaningful cross-model comparisons in LLM-based research. This approach reflects the study’s primary aim: to evaluate and compare the performance of multiple LLMs in generating patient education content for mitral valve surgery.

### Limitations

Although the independent and double-blind evaluation of the data by two expert surgeons strengthens the assessment method and analysis, this study has certain limitations that should be acknowledged. The findings reported herein should be interpreted within the context of a limited question set (seven items) and should be considered exploratory rather than definitive comparisons of model performance. First, the responses were evaluated by two experts, and while inter-rater reliability was high (ICC = 0.85), human judgment inherently carries some degree of subjectivity, which cannot be entirely eliminated even with strong inter-evaluator agreement. Second, the evaluation was conducted only on responses in English; model performances in other languages could not be assessed. Third, the examined topic was limited to MVS, and model performance may vary for other diseases; therefore, making a general characterization of LLMs may not always be fully accurate. In addition, the study analyzed specific versions of the LLMs at a single time point. As LLMs are updated frequently, their performance could change over time, potentially altering accuracy, completeness, and readability outcomes in future assessments. Fourth, cultural differences among patients using LLMs may exist, and behavioral outcomes such as patient experience and satisfaction were not included in the evaluation, which can be deemed as another limitation. Finally, a relatively small number of questions was used for model evaluation. The use of only seven items may have contributed to a ceiling effect, with high scores across models and limited score dispersion, which may reduce the practical significance of observed statistical differences. Differences in future versions of LLMs may also affect the outcomes. Additionally, the original LLM-generated outputs were not systematically archived at the time of the study. Given the continuously evolving nature of large language models, exact replication of the original responses is not feasible, which may limit reproducibility. Moreover, response consistency across repeated queries was not evaluated and represents an area for future research.

## Conclusion

In conclusion, the role of large language models (LLMs) in patient education and health information delivery has been increasingly discussed in the healthcare literature. In this study, ChatGPT-4o and Gemini 2.5 Pro Preview demonstrated higher accuracy and completeness in responses to patient-related queries on mitral valve surgery, while Claude 3.7 Sonnet showed superior readability. These findings reflect differences in model performance across selected prompts rather than demonstrated patient outcomes. Accordingly, these results should not be interpreted as evidence of clinical effectiveness or patient benefit. Within these limitations, LLMs may be considered to have potential utility as adjunct tools for enhancing the clarity and accessibility of patient education materials, only when used under appropriate clinician oversight. Future studies incorporating patient-centered evaluations and accounting for variables such as age, educational background, and digital literacy are needed to more fully assess their role in clinical practice.

## Supplementary Information

Below is the link to the electronic supplementary material.

Supplementary Material 1

## Data Availability

The data that support the findings of this study are available from the corresponding author upon reasonable request. The dataset consists of LLM-generated responses and corresponding evaluation scores.
