# Supplementary material for: Performance of large language models in mitral valve surgery patient education: a comparative analysis
Source: BMC Med Inform Decis Mak. 2026 Jun 29;26:352. doi: 10.1186/s12911-026-03662-3 (PMC13579825; doi:10.1186/s12911-026-03662-3)
Supplement: Supplementary file 1 [file 12911_2026_3662_MOESM1_ESM.pdf]

This site uses cookies to bring you the best experience. **Find out more (/cookiepolicy.aspx)** Continue

Member's Login (../../../../members/default.aspx)

Patients (../../../../patients.aspx)

Cardiac (Heart) (/patients/heart.aspx)

Procedures (/patients/heart/procedures/)

Aortic Arch Repair (/patients/heart/procedures/23/aortic_arch_repair)

Aortic Root Repair (/patients/heart/procedures/22/aortic_root_repair)

Aortic Valve Surgery (/patients/heart/procedures/19/aortic_valve_surgery)

Atrial Fibrillation Surgery (/patients/heart/procedures/21/atrial_fibrillation_surgery)

Coronary artery bypass grafts (/patients/heart/procedures/18/coronary_artery_bypass_grafts)

Heart Transplant (/patients/heart/procedures/38/heart_transplant)

Mitral Valve Surgery (/patients/heart/procedures/20/mitral_valve_surgery)

Pericardectomy (/patients/heart/procedures/25/pericardectomy)

Thoraco-Abdominal Repair (/patients/heart/procedures/24/thoracoabdominal_repair)

Transcatheter Aortic Valve Implant (TAVI) (/patients/heart/procedures/26/transcatheter_aortic_valve_implant_tavi)

Glossary (/patients/heart/glossary/)

Patient Journey (/patients/heart/patient_journey.aspx)

Outcome Data (/patients/heart/outcome_data/default.aspx)

Thoracic (Lungs) (/patients/lungs.aspx)

Mitral Valve Surgery

Mitral valve surgery is a type of open-heart surgery used to treat problems with the heart's mitral valve. The mitral valve is a pair of flaps in the heart that stops blood flowing the wrong way (back towards your lungs). Surgery is often the only way that the valve can function normally and prevent your heart from deteriorating. Mitral valve surgery involves repairing or replacing the faulty or damaged valve. If the valve is replaced, then your diseased valve is removed and replaced with a new one made from synthetic materials (mechanical) or animal tissue (tissue).

Why do I need surgery?

What does the anaesthetic involve?

What does the surgery involve?

What happens after surgery?

What are the benefits and risks?

What should I do when I go home?

What are the alternatives to surgery?

Home (../../../../Default.aspx)

About SCTS (../../../../about_scts.aspx)

Patients (../../../../patients.aspx)

Professionals (../../../../professionals.aspx)

Membership (../../../../membership.aspx)

SCTS Annual Meeting (../../../../scts_annual_meeting.aspx)

Who We Are (/about_scts/who_we_are.aspx)

News (/news)

SCTS Spotlight (https://scts.org/about_scts/scts_spotlight.aspx)

Noticeboard (/about_scts/noticeboard.aspx)

Executive (/about_scts/committees/18/executive_committee/standalone)

SCTS Vacancies (/about_scts/scts_vacancies.aspx)

Our History (/about_scts/our_history.aspx)

Constitution (/about_scts/constitution.aspx)

Membership Application (/join/default.aspx)

Contact Options (/about_scts/contact_options.aspx)

Merchandise (/about_scts/merchandise/)

Cardiac (Heart) (/patients/heart.aspx)

Thoracic (Lungs) (/patients/lungs.aspx)

Congenital (Children) (/patients/congenital.aspx)

Support Groups (/patients/support_groups.aspx)

Find a Hospital (/patients/hospitals/)

Find a Consultant (/patients/consultants/)

Donate (/patients/donate.aspx)

Useful Information (/patients/useful_information.aspx)

Surgical Sub-specialities (/surgical_sub_specialities.aspx)

Medical Students (/medical_students.aspx)

Nursing and AHP (/professionals/surgical_sub_specialities/nursing_and_ahp.aspx)

Surgical Training (/professionals/surgical_training.aspx)

Left-Handed (https://scts.org/left-handed.aspx)

Research (/professionals/research.aspx)

Reports & Resources

Equality, Diversity and Inclusion (/professionals/equality_diversity_and_inclusion.aspx)

Women in Cardiothoracic Surgery Network (/professionals/women_in_cardiothoracic_surgery_network.aspx)

Committees (/professionals/committees/)

Board of Representatives (/professionals/committees/14/board_of_representatives/standalone)

Innovation (/professionals/innovation.aspx)

Member wellbeing (/professionals/member_wellbeing.aspx)

Membership Benefits (/membership/membership_benefits.aspx)

Members' Area (/members/default.aspx)

SCTS Annual Meeting (/upcoming_meeting_and_scts_university/default.aspx)

Frequently Asked Questions (/scts_annual_meeting/annual_meeting_faqs.aspx)

Code of Conduct (https://scts.org/code_of_conduct.aspx)

Previous Meetings (/scts_annual_meeting/previous_meetings.aspx)

Videos (/scts_annual_meeting/videos_.aspx)

Exhibition & Sponsorship (/sponsorship.aspx)

Accommodation (https://scts.org/scts_annual_meeting/accommodation.aspx)

Registration (https://scts.org/annual_meeting_registration_.aspx)

Scientific Programme (https://scts.org/annual_meeting_programme.aspx)

Society for Cardiothoracic Surgery
in Great Britain and Ireland

(https://www.instagram.com/s_c_t_s/)

(https://www.linkedin.com/company/the-society-for-cardiothoracic-surgery-in-great-britain-and-ireland/)

(https://www.facebook.com/SCTSGBI/)

(https://twitter.com/sctsuk)

Society for Cardiothoracic Surgery in Great Britain & Ireland

38-43 Lincoln's Inn Fields, London, WC2A 3PE

Registered Charity No. 1113536 Company No. 05685735 VAT No. 576 9529 77

Disclaimer (../../../../disclaimer.aspx)

Terms (../../../../terms.aspx)

Cookie Policy (../../../../cookiepolicy.aspx)

Log In (/login.aspx)

Designed & Developed by LightMedia (https://www.light-media.co.uk)
